# Retrospective Evaluation of Mucormycosis Cases at a Tertiary Care Center Between 2000 and 2020 in Türkiye

**DOI:** 10.1007/s11046-026-01073-6

**Published:** 2026-04-29

**Authors:** Hazel Öztürk Belik, Yasemin Heper, Esra Kazak, Emel Yılmaz, Beyza Ener, Halis Akalın

**Affiliations:** 1 Faculty of Medicine, Infectious Diseases and Clinical Microbiology, Bursa Uludag University, Bursa, Türkiye; 2 Faculty of Medicine, Medical Microbiology, Medical Mycology, Bursa Uludag University, Bursa, Türkiye

**Keywords:** Mucormycosis, Invasive fungal infection, Amphotericin B, Posaconazole

## Abstract

Mucormycosis ranks third among invasive mycoses after Candidiasis and Aspergillosis and is associated with high mortality. Its incidence has increased with the rising number of immunosuppressed patients. In developing countries, the most common predisposing factor is uncontrolled diabetes mellitus (DM), whereas in developed countries it is immunosuppressive conditions. To examine local epidemiological data, predisposing factors, diagnostic and therapeutic options and survival in our center eighty-six adult mucormycosis patients between 2000 and 2020 were retrospectively analyzed. Thirty-nine (45.35%) were male, 47 (54.65%) were female, and the median age was 52 years (IQR, 42.5–62). The most frequent clinical presentation was sinus involvement, observed in 77 cases (89.53%). Of these, 38 (44.18%) were sinonasal, 5 (5.81%) sinoorbital, 9 (10.47%) rhinocerebral, and 25 (29.07%) rhino-orbito-cerebral mucormycosis. The remaining 9 cases (10.47%) had pulmonary mucormycosis. Predisposing factors included hematologic diseases in 51 patients (59.30%), DM in 33 (38.37%), solid organ transplantation (SOT) in 7 (8.14%), and solid organ malignancy in 7 (8.14%). The most common symptoms were fever (70.93%), swelling around the eyes and face (51.16%), pain (40.7%), erythema (34.88%), nasal discharge (30.23%), and headache (25.58%). Most frequent physical examination findings were necrotic lesions in the oral cavity and sinuses (87.21%), ophthalmoplegia (29.07%), ptosis (26.74%), vision loss (25.58%), and proptosis (22.09%). In all cases, amphotericin B formulations were preferred as initial therapy. Patients who received combination therapy (n = 14), 13 used posaconazole (POS) + liposomal amphotericin B (L-AmB) and one patient used itraconazole (ITC) + L-AmB. POS was administered to 14 patients receiving sequential oral therapy. The median duration of amphotericin B therapy was 46.5 days (IQR, 14.7–84.5), and the median total duration of antifungal therapy was 46.5 days (IQR, 14.7–90.3). The most common adverse effect of amphotericin B formulations was hypokalemia [L-AmB 68.75%, amphotericin B deoxycholate (AmB-D) 54.84%, amphotericin B lipid complex (ABLC) 50%]. At least one surgical intervention was performed in 74.42% of cases. The 12-week mortality rate was 48.84%, overall mortality rate was 61.63%. In analysis advanced age [12-week: OR: 1.04 (95% CI: 1.01–1.07), *p* = 0.011; overall: OR: 1.04 (95% CI: 1.01–1.07), *p* = 0.026 respectively], coexistence of both hematologic disease and DM [12-week OR: 5.73 (95% CI: 1.16–28.33), *p* = 0.032] associated with mortality. No significant difference was observed in 12-week mortality between surgical and non-surgical patients (*p* = 0.107). In contrast, overall mortality was significantly higher in the non-surgical group (81.8% vs. 54.7%, *p* = 0.024). In univariable logistic regression analysis, surgical intervention was associated with a 73.2% reduction in the odds of overall mortality (OR: 0.268, 95% CI: 0.082–0.882, *p* = 0.030). In the multivariable logistic regression analysis, age was the only variable significantly associated with 12-week and overall mortality (respectively, *p* = 0.004 and *p* = 0.026). Each one-year increase in age was associated with an OR of 1.05 (95% CI: 1.02–1.09) for 12-week mortality and 1.04 (95% CI: 1.01–1.07) for overall mortality. In conclusion; our study showed that despite advanced diagnostic methods and antifungals, mortality remained high, sinus involvement was the most frequent involvement, surgical debridement increased survival, but increasing age was associated with poor prognosis.

## Introduction

Mucormycosis is a rare invasive fungal infection with high morbidity and mortality rates [1]. It progresses with vascular invasion and tissue necrosis, leading to an acute or subacute clinical picture [2, 3]. Among invasive mycoses, it ranks third after Candidiasis and Aspergillosis [4]. It particularly causes acute angioinvasive infection in immunocompromised patients [3]. Due to the increasing number of immunocompromised individuals, better awareness, and the development of new diagnostic tools its incidence is also rising [5–7]. Because of its rarity and diagnostic difficulties, its incidence is not precisely known [6, 8]. The incidence rate globally varies from 0.005 to 1.7 per million population [9].

Species of *Mucorales* are widespread in nature worldwide; however, because of their low virulence and requirement for specific growth conditions, they generally cause secondary infections [10, 11]. Well-known predisposing factors include hematologic malignancies, prolonged neutropenia, uncontrolled diabetes mellitus (DM), diabetic ketoacidosis (DKA) and other causes of metabolic acidosis, solid organ malignancies, bone marrow transplantation (BMT) and solid organ transplantation (SOT), long-term or high-dose corticosteroid use, deferoxamine therapy, prolonged voriconazole (VRC) therapy, intravenous drug use, HIV/AIDS, malnutrition, and prematurity [2, 3, 11, 12]. In developed countries, they are most frequently reported in patients with hematologic malignancies, while in developing countries they are commonly associated with uncontrolled DM and trauma [13–15].

The pathogenesis in immunocompetent hosts is primarily driven by the direct inoculation of fungal spores into compromised integumentary or mucosal barriers. This mode of acquisition is often precipitated by extensive burns or major trauma [6, 11, 12]. Such mechanisms are strikingly evident in the aftermath of natural disasters like floods, storms, earthquakes etc. —as seen in the clusters following the 2004 Indian Ocean tsunami [16] and the 2011 Joplin tornado [17]— as well as in complex combat-related injuries [18]. Beyond environmental inoculation, healthcare-associated transmission remains a significant concern, with iatrogenic outbreaks frequently linked to contaminated medical supplies such as catheters, surgical adhesives, hospital linens and tongue depressors. Beyond direct contact with contaminated materials, the hospital environment itself poses risks; investigations into increased airborne fungal loads are essential, particularly during hospital renovation or construction activities near patient care units. Additionally, hospital water systems should be recognized as a potential reservoir for these opportunistic fungi [19].

Due to its rapid course, urgent intervention is required once clinical suspicion arises. Delay in treatment increases mortality. Improving survival rates demands rapid diagnostic and therapeutic strategies with a multidisciplinary approach [6, 20].

The clinical form of mucormycosis depends on the patient’s underlying disease and the route of fungal entry. The most frequent clinical presentations are rhinocerebral, pulmonary, disseminated, gastrointestinal, and cutaneous involvement [4].

The hallmark of mucormycosis is angioinvasion. The result is thrombosis and tissue necrosis, impairing the penetration of antifungal agents into the site of infection, thereby reducing drug efficacy. Therefore, removal of necrotic tissue and fungal burden is required [2, 6, 21]. Since debridement of necrotic tissue is often disfiguring, major reconstructive surgery may be required once the patient survives the acute phase [2].

Amphotericin B, posaconazole (POS), and isavuconazole (ISA) are the most potent antifungal agents against mucormycosis pathogens [22, 23]. Both the European Conference on Infections in Leukemia (ECIL) [24] and the European Confederation of Medical Mycology (ECMM) guidelines [6] recommend lipid formulations of amphotericin B as initial therapy.

In the ECMM guidelines, POS and ISA are recommended intravenously as initial therapy [instead of liposomal amphotericin B (L-AmB)] in cases of renal failure [6]. In the ECIL guidelines, however, POS is recommended only for salvage and maintenance therapy [24]. Both guidelines recommend L-AmB and POS combination therapy as salvage treatment [6, 24].

With the increasing number of immunosuppressed patients, changes have occurred over the years in the number of mucormycosis cases and the distribution of underlying diseases. This study aimed to examine local epidemiological data, predisposing factors, diagnostic and therapeutic options, and survival in our center.

## Materials and Methods

The study was conducted at Bursa Uludag University Hospital, an 800-bed tertiary institution, which serves as a specialized center for hematological and oncological malignancies, as well as bone marrow and solid organ transplantation, nearby all other pediatric and adult specialities. Between 2000 and 2020, the median annual number of inpatient admissions was 51,980 (IQR, 26,692.5–100,374.5). During this period, data from 86 adult patients diagnosed with mucormycosis were retrospectively analyzed through the electronic medical record system, following the approval of the ethics committee (Approval date: July 28, 2021; Approval number: 2021—10/5).

The diagnosis of mucormycosis was established based on clinical findings supported by at least one of microbiological, pathological, or radiological evidence. Demographic and clinical characteristics of the patients were collected. Age, sex, underlying disease, medications, symptoms, physical examination findings, culture and pathology results, imaging findings, diagnostic treatments, and survival were evaluated.

Except for one patient from whom a sample could not be obtained due to thrombocytopenia, at least one of direct microscopy, culture, or histopathological examination was performed in 85 cases. Endoscopic sinus biopsy, bronchoscopic biopsy, surgical material, sputum, abscess, and pleural fluid samples were obtained, treated with 10% KOH, and examined under direct microscopy. The presence of broad, irregular, non-septate or rarely septate hyphae was accepted as supportive of the diagnosis. Growth of fungi of the order *Mucorales* on Sabouraud dextrose agar was considered sufficient, but no genus- or species-level identification was performed. In cytological examination, hyphal structures were identified with Papanicolaou and PAS staining; in histopathological examination, hyphal structures, vascular invasion, necrosis, inflammation, and perineural invasion findings were identified with HE and PAS staining as diagnostic features. Cytological examination was performed on bronchoalveolar lavage fluid, while histopathological examination was conducted on biopsy specimens.

Cases were classified as proven when histopathological examination of biopsy specimens demonstrated positivity or when cultures or direct microscopic examinations of samples obtained from sterile tissues or body fluids were positive. Probable cases were defined by the detection of positive culture or direct microscopic examination results from non-sterile tissue samples in the presence of compatible clinical findings. Possible cases were identified when clinical and radiological findings supported the diagnosis despite the absence of histopathological or microbiological confirmation [25].

In this study, the conformity of continuous variables to normal distribution was tested with the Shapiro–Wilk test. Variables not conforming to normal distribution were expressed as median (IQR, Q1–Q3) values. For comparisons between two groups, the Mann–Whitney U test was used when the normality assumption was not met; in cases with more than two groups, the Kruskal–Wallis test was applied. Following a significant Kruskal–Wallis test result, subgroup analyses were conducted using the Dunn–Bonferroni test. Categorical variables were expressed as n (%). Comparisons of categorical variables between groups were performed using Pearson’s chi-square test, Fisher’s exact chi-square test, and the Fisher–Freeman–Halton test. Factors associated with 12-week and overall mortality were analyzed using univariable and multivariable logistic regression analysis. The forward selection method was employed for variable selection. The multivariable logistic regression model demonstrated a good fit to the data (Hosmer and Lemeshow test, p = 0.198 and p = 0.770) and was found to be statistically significant (*p* < 0.001 and *p* = 0.022). Statistical analyses were performed using SPSS (IBM Corp. Released 2012. IBM SPSS Statistics for Windows, Version 21.0. Armonk, NY: IBM Corp.), and the significance level for type I error was set at *p* < 0.05.

## Results

Of the 86 cases, 39 (45.35%) were male and 47 (54.65%) were female. The median age of the cases was 52 years (IQR, 42.5–62). Patients most frequently presented in autumn (n = 36, 41.86%) and summer (n = 31, 36.05%).

Regarding clinical forms, sinus involvement was identified in 77 cases (89.53%) and pulmonary involvement in 9 (10.47%). Among the 77 cases with sinus disease, 38 (44.18%) were sinonasal, 25 (29.07%) rhino-orbito-cerebral, 9 (10.47%) rhinocerebral, and 5 (5.81%) sinoorbital mucormycosis. Of the 9 cases with pulmonary involvement, only 1 (1.16%) had chest-wall invasive disease; the remaining 8 (9.31%) had localized pulmonary mucormycosis.

All patients had at least one predisposing factor. Hematologic disease was present in 51 cases (59.30%), DM in 33 (38.37%), and solid organ malignancy in 7 (8.14%). There were 7 patients (8.14%) who had undergone SOT and 1 (1.16%) who had undergone allogeneic BMT (Table 1). Eleven patients (12.79%) had both a hematologic disease and DM. Our study was limited to the first year of the COVID-19 pandemic, and no COVID-19-associated mucormycosis (CAM) cases were identified in this cohort.

**Table 1:** Distribution of underlying diseases

	n* (%)
Metabolic diseases	46 (53.5)
Diabetes mellitus	33 (38.4)
Hyperthyroidism	1 (1.2)
Hypothyroidism	2 (2.3)
Hyperlipidemia	6 (7.0)
Hypopituitarism	1 (1.2)
Osteoporosis	1 (1.2)
Polycystic ovary syndrome	1 (1.2)
Acromegaly	1 (1.2)
Hematologic diseases	51 (59.3)
Acute myeloid leukemia	29 (33.7)
Acute lymphoblastic leukemia	14 (16.3)
Non-Hodgkin lymphoma	3 (3.5)
Aplastic anemia, autoimmune hemolytic anemia, Evans syndrome	3 (3.5)
Myelodysplastic syndrome	2 (2.3)
Bone marrow transplantation	
Allogeneic bone marrow transplantation	1 (1.2)
Solid organ malignancy	7 (8.1)
Lung cancer	2 (2.3)
Bladder cancer	1 (1.2)
Gastric cancer	1 (1.2)
Hepatocellular carcinoma	1 (1.2)
Ovarian cystadenoma	1 (1.2)
Pituitary macroadenoma	1 (1.2)
Solid organ transplantation	
Kidney transplant	7 (8.1)
Chronic renal failure	2 (2.3)
Cardiac diseases	
Hypertension	24 (27.9)
Coronary artery disease	6 (7.0)
Atrial fibrillation	2 (2.3)
Heart failure	3 (3.5)
Rheumatologic diseases	3 (3.5)
Systemic lupus erythematosus	1 (1.2)
Microscopic PAN	1 (1.2)
Rheumatoid arthritis	1 (1.2)
Pulmonary diseases	6 (7.0)
Asthma	2 (2.3)
COPD	4 (4.7)
Liver diseases	4 (4.7)
Cirrhosis	2 (2.3)
Chronic hepatitis C	1 (1.2)
Hepatic steatosis	1 (1.2)
Benign prostatic hyperplasia	1 (1.2)
Alzheimer’s disease	1 (1.2)
Toxic epidermal necrolysis	1 (1.2)

^*^n = *86*

**Fig. 1:**
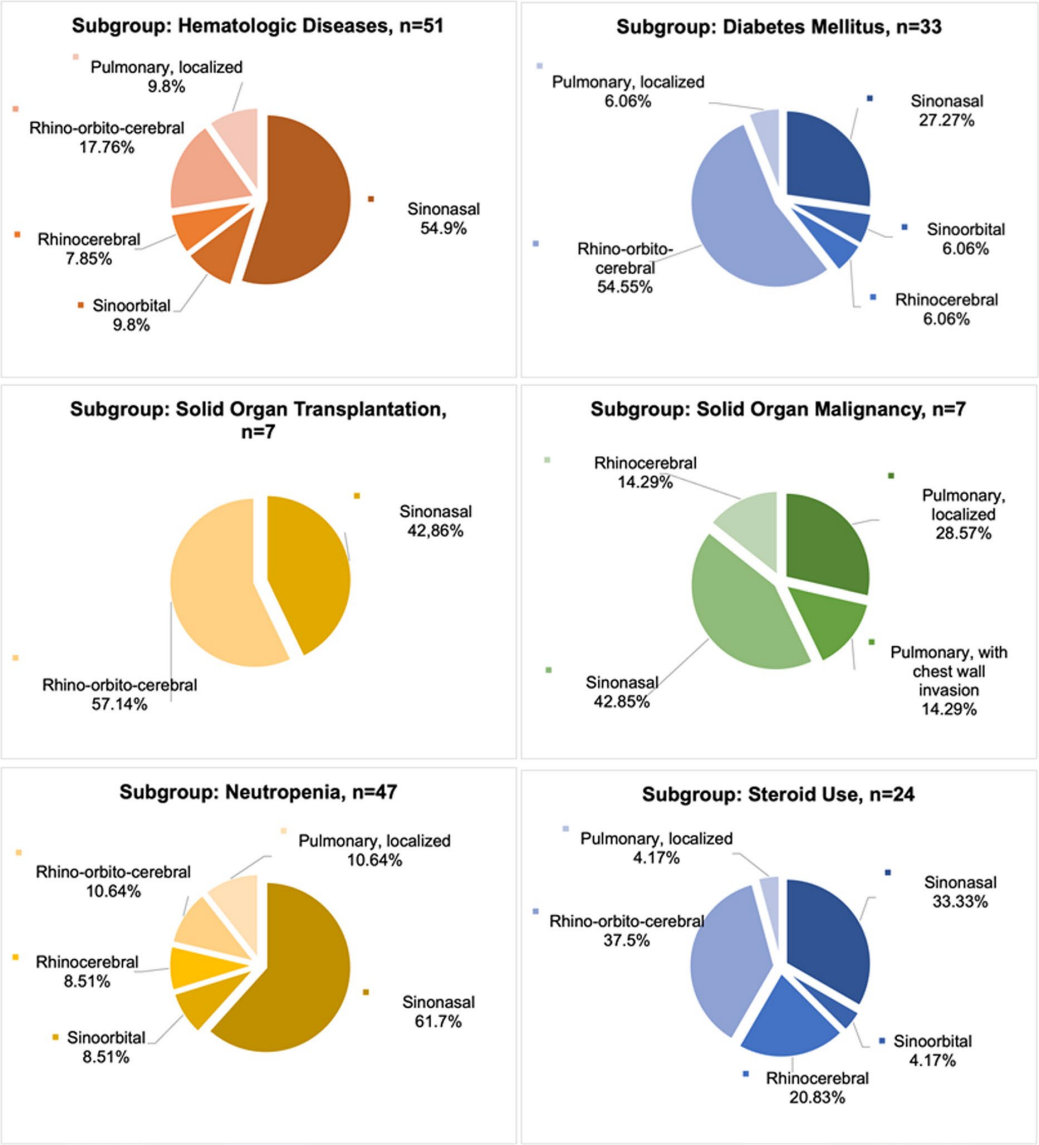
Distribution of clinical forms by underlying conditions

Differences in the involved sites according to underlying diseases were observed (Fig. 1).

When 33 cases with a diagnosis of DM were analyzed, the median blood glucose level at admission was 339 (IQR, 287–428). Eleven patients (33.4%) presented with DKA. In 5 patients (15.15%), DM was diagnosed after the diagnosis of mucormycosis. HbA1c assessment was limited to 17 patients, with a median of 7.9% (IQR 6.8–11.2).

When patients receiving immunosuppressive therapy were analyzed, it was observed that 46 received chemotherapy for malignancy, 24 were on corticosteroids. Other therapies included calcineurin inhibitors (n = 9), intravenous immunoglobulin (n = 6), anti-thymocyte globulin (n = 5), mTOR inhibitors (n = 3) and rituximab (n = 1). There were also patients who received these treatments in various combinations. Among 46 patients receiving chemotherapy for malignancy, 28 had acute myeloid leukemia (AML), 13 had acute lymphoblastic leukemia, 3 had non-Hodgkin lymphoma, and 2 had solid organ tumors treated with various chemotherapies.

Among the 47 neutropenic patients, the median duration of neutropenia was 4 weeks (IQR, 2–4) before the diagnosis of mucormycosis. In 44 of these patients, neutropenia was associated with chemotherapy for malignancy. In the remaining three patients, neutropenia was due to methotrexate therapy for rheumatoid arthritis, aplastic anemia, and AML, respectively. In neutropenic patients, sinonasal involvement was observed in 61.7%, while in steroid users, rhino-orbito-cerebral involvement was observed in 37.5% (Fig. 1).

The most frequent presenting symptoms were fever, periorbital and facial swelling, pain, erythema, nasal discharge, and headache. On physical examination, necrotic lesions of the oral cavity and sinuses and ocular findings were the most common (Table 2).

**Table 2:** Frequency of presenting symptoms and physical examination findings

Symptoms	n* (%)	Findings	n* (%)
Fever	61 (70.9)	Necrosis in oral cavity, nose, and sinuses	75 (87.2)
Periorbital / facial swelling	44 (51.2)	Ophthalmoplegia	25 (29.1)
Periorbital / facial pain	35 (40.7)	Ptosis	23 (26.7)
Periorbital / facial erythema	30 (34.9)	Vision loss	22 (25.6)
Nasal discharge	26 (30.2)	Proptosis	19 (22.1)
Headache	22 (25.6)	Anisocoria	13 (15.1)
Facial numbness	10 (11.6)	Chemosis	11 (12.8)
Nasal congestion	9 (10.5)	Peripheral facial paralysis	9 (10.5)
Cough – sputum	9 (10.5)	Hemiplegia – hemiparesis – paraplegia	7 (8.1)
Vision loss	8 (9.3)	Purulent discharge in oral cavity, nose, and sinuses	7 (8.1)
Nasal–oral ulcer	6 (7.0)	Rales	5 (5.8)
Dizziness	5 (5.8)	Palatal perforation – fistula	5 (5.8)
Dyspnea	4 (4.7)	Dysphagia	4 (4.7)
Diplopia	4 (4.7)	Pulmonary necrosis	4 (4.7)
Nausea – vomiting	3 (3.5)	Speech disorder	4 (4.7)
Sore throat	2 (2.3)	Central facial paralysis	4 (4.7)
Loss of consciousness	2 (2.3)	Confusion	3 (3.5)
Ocular discharge	1 (1.2)	Deviation of the tongue	3 (3.5)
Regurgitation of food through the nose	1 (1.2)	Babinski sign	3 (3.5)
		Dyspnea	2 (2.3)
		Bronchial secretions	2 (2.3)
		Other (anosmia, suprasellar abscess, hemoptysis, vocal cord paralysis, bronchial fistula, empyema)	6 (7.0)

^*^n = *86*

A total of 86 mucormycosis cases were evaluated in the present study, among which 59 were classified as proven, 21 as probable and 6 as possible. In 85 patients, at least one of direct microscopy, culture, or pathological examination was performed, while in one patient, an invasive tissue sampling was not possible due to thrombocytopenia. Direct microscopic examination was performed in 81 patients, positive in 62 (76%); culture in 78 patients, positive in 49 (62.83%); histopathology in 74 patients, positive in 59 (79.73%). Direct microscopy and pathology were simultaneously positive in 45 patients, and culture and pathology in 35 patients. Identification at the genus- or species-level could not be achieved.

For diagnosis, staging, and treatment response evaluation, paranasal–orbital–cranial CT was performed in 70 cases, cranial–orbital MRI in 55 cases, and thoracic CT in 9 cases. All patients with pulmonary involvement underwent imaging. However, among the 77 cases with sinus involvement, imaging was incomplete for some patients due to poor clinical status or localized disease; specifically, seven did not undergo CT and 22 did not undergo MRI. An additional six patients with underlying hematologic or oncologic malignancies died within three weeks of diagnosis before any imaging could be performed. Radiological findings are presented in Table 3.

**Table 3:** Radiological findings

Cranial – Paranasal – Orbital CT (n = 70)	n (%)
Mucosal edema	63 (90.0)
Soft tissue edema	28 (40.0)
Loss of aeration in sinuses	24 (34.3)
Bone erosion	22 (31.4)
Abscess	4 (5.7)
Parenchymal hemorrhage	2 (2.9)

Cranial – Orbital MRI (n = 55)	n (%)
Cavernous sinus involvement	18 (32.7)
Infarction and thrombosis	15 (27.3)
Orbital invasion	15 (27.3)
Dural enhancement – Cerebritis – Cerebellitis – Brainstem involvement – Abscess	12 (21.8)
Periorbital inflammation	10 (18.2)

Thorax CT (n = 9)	n (%)
Ground-glass opacity – Consolidation	9 (100.0)
Pleural effusion	6 (66.7)
Nodule	4 (44.4)
Halo – Reverse halo	3 (33.3)
Lymph node	3 (33.3)
Cavity	3 (33.3)

Prior to the diagnosis of mucormycosis, 32 patients (37.21%) had been receiving antifungal prophylaxis, all of whom had hematologic malignancies. Twelve patients (13.95%) received fluconazole (FLC) (10 oral, 2 IV) and 20 (23.26%) received oral POS prophylaxis.

All patients initially received amphotericin B formulations [amphotericin B deoxycholate (AmB-D), L-AmB, amphotericin B lipid complex (ABLC)]. Of the 14 patients who later received combination therapy, 13 were given POS and 1 itraconazole (ITC); 14 patients (16.28%) received oral POS alone as maintenance therapy.

The median duration of amphotericin B therapy was 46.5 days (IQR, 14.7–84.5), and the median total duration of antifungal therapy was 46.5 days (IQR, 14.7–90.3). Among patients with short treatment duration (≤ 60 days, n = 53), 43 (81.13%) died. In the majority of deceased patients (n = 33), the brevity of treatment duration was due to early mortality resulting from the severity of their underlying diseases. Of these 43, 28 had hematologic disease and 17 had DM, with 9 having both. Among patients with short treatment but not deceased (n = 10), early surgery and localized sinus disease were present.

Of 31 patients initially treated with AmB-D, 3 continued, while 26 were switched to L-AmB and 2 to ABLC due to side effects or lack of response. Reasons for switching included allergic reactions (n = 10), elevated urea–creatinine (n = 9), lack of clinical/radiological response (n = 7), elevated AST–ALT (n = 1), and drug unavailability (n = 1). Median time to treatment switch was 8 days (IQR, 3–21.5). Of the 2 patients starting ABLC, 1 was switched to L-AmB due to allergy. None of the 53 patients initially treated with L-AmB required a change. In total, 80 patients were treated with L-AmB, 3 with AmB-D, and 3 with ABLC. Among 80 patients on L-AmB, dose escalation was required in 15 due to lack of response, and dose reduction in 8 due to elevated urea-creatinine and in 1 due to elevated AST–ALT.

The majority of our cases received L-AmB at a dosage of 5 mg/kg. However, dose reductions or treatment interruptions were required in several instances due to various reasons, such as hypokalemia and impairment of renal or hepatic functions. In a limited number of cases, the dosage was escalated to levels of 7–10 mg/kg. These dose modifications were permanent in some patients and transient in others. Furthermore, the duration and magnitude of these adjustments varied for each patient. Due to this significant heterogeneity and the non-permanent nature of these changes, a standardized dose-based evaluation could not be performed. Overall, 14 patients (16.28%) required combination therapy due to lack of response. Of these, 12 had started with L-AmB and 2 with AmB-D later switched to L-AmB. Combination therapy included L-AmB with POS in 13 and L-AmB with ITC in 1. Median duration of combination therapy was 57 days (IQR, 33.8–101.3).

Adverse effects of AmB-D (n = 31) included hypokalemia in 17, allergic reactions in 12, and elevated urea–creatinine in 10. Among patients on L-AmB (n = 80), 55 had hypokalemia, 15 had elevated urea–creatinine, and 11 had elevated AST–ALT. Both ABLC patients had hypokalemia; one also developed rash and pruritus. Overall, hypokalemia was the most common adverse effect of amphotericin B formulations (L-AmB 68.75%, AmB-D 54.84%, ABLC 50%). In total, 62 patients developed hypokalemia, while elevated blood urea and creatinine levels were observed in 24 patients.

Surgical intervention was performed in 64 patients (74.42%). In the remaining 22, indications were absent (n = 11), poor clinical status prevented intervention (n = 8), or patients refused (n = 3). Among operated patients, 50 underwent endoscopic sinus surgery, 14 open sinus surgery, 2 thoracotomy, and 2 intracranial abscess excision. Orbital exenteration was performed in 9 cases, and reconstruction in 7. Median time to first intervention was 1 day (IQR, 1–3), with a median of 1 intervention (IQR, 1–2) per patient.

During hospitalization, all patients developed at least one bacterial or viral co-infection. The most common were pneumonia (66.28%), bacteremia (39.53%), and urinary tract infection (23.26%). A fungal co-infection was found in 32 patients (28 aspergillosis, 3 candidiasis, 2 fusariosis). Of these, 27 (84.38%) had hematologic disease (Table 4). Regarding fungal co-infections, the causative agents were identified as follows: *A. fumigatus* in 8 samples (4 sinonasal, 4 lower respiratory tract), *A. flavus* in 8 samples (6 sinonasal, 2 lower respiratory tract), *A. niger* in 2 samples (sinonasal), and *A. terreus* in 2 samples (1 sinonasal, 1 lower respiratory tract). Furthermore, *Fusarium* spp. were isolated in 2 samples (1 sinonasal, 1 blood), while *C. krusei* and *C. albicans* were each detected in 1 blood sample. In cases where no microbial growth was obtained, the diagnosis of invasive fungal infection was established based on clinical and radiological findings combined with galactomannan positivity in serum or bronchoalveolar lavage samples.

**Table 4:** Distribution of co-infections by number of cases

Co-infection	n (%)
Pneumonia	57 (66.3)
Bacteremia	34 (39.5)
Fungal co-infection	32 (37.2)
Urinary tract infection	20 (23.3)
Mucositis	11 (12.8)
Skin and soft tissue infection	11 (12.8)
Catheter infection	10 (11.6)
Sinusitis	9 (10.5)
Herpes labialis	7 (8.1)
Intra-abdominal infection	6 (7.0)
Central nervous system infection	5 (5.8)
Genital system infection	4 (4.7)
CMV viremia	3 (3.5)
Skull base osteomyelitis	3 (3.5)
Pulmonary tuberculosis	2 (2.3)
Endocarditis	1 (1.2)

^*^n = *86*

Among the 32 patients with fungal co-infections, antifungal therapy remained unchanged in 17 cases, 15 of whom additionally received combination therapy with caspofungin (CAS), ITC, or VRC. A total of 18 patients with fungal co-infections died.

Median hospitalization was 72 days (IQR 38–118.5). Intensive care unit (ICU) admission occurred in 18 patients (20.93%), all of whom died. Median ICU stay was 16.5 days (IQR, 5.5–34.5).

**Fig. 2:**
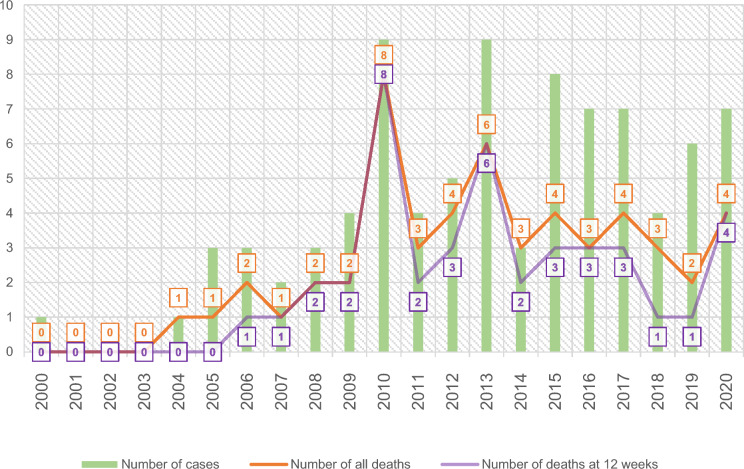
Trends in case numbers and deaths by year

**Fig. 3:**
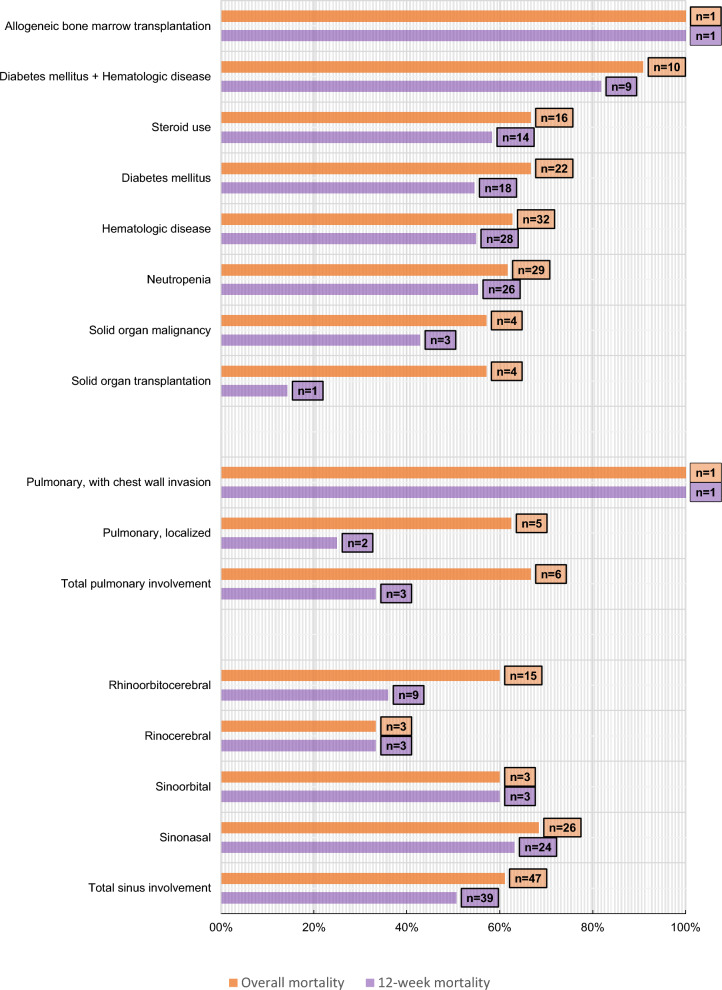
Mortality rates by underlying diseases and clinical forms

In total, 53 of 86 patients died (61.63%). In 22 (41.51%), the cause of death was progression of mucormycosis; in 31, it was primary disease or secondary infections. The median time from diagnosis to death was 3 weeks (IQR, 2–8). The 12-week mortality was found to be 48.84% (n = 42). Also 12-week mortality rate was 54.9% in hematologic diseases (overall 62.75%), 54.55% in DM (overall 66.67%), and 42.86% in solid organ malignancy (overall 57.14%). Trends in case numbers and deaths by year are shown in Fig. 2, while mortality rates according to underlying diseases and clinical forms are presented in Fig. 3.

In the two-group comparison analysis, the median age of deceased patients was significantly higher than that of survivors (12-week: 50.5 vs. 34.5, *p* = 0.008; overall: 56 vs. 48, *p* = 0.015). Mortality was higher in patients with both hematologic disease and DM (12-week: 21.42% vs 4.5%, *p* = 0,019; overall 18.87% vs 3.03%, *p* = 0.045). Presence of DM alone, hematologic disease alone, or presentation with DKA was not statistically associated with 12-week or overall mortality (*p* > 0.05). Neutropenia, steroid use, solid organ malignancy, SOT, and BMT were also not associated with 12-week or overall mortality (*p* > 0.05). There were no significant differences in symptoms, physical findings, or involved sites between 12-week and overall mortality groups (*p* > 0.05).

Although all patients initially received amphotericin B formulations (L-AmB 53, AmB-D 31, ABLC 2), 29 (33.7%) required a change. Prognosis did not differ significantly between patients treated continuously with L-AmB and those who underwent a treatment switch (*p* > 0.05). Similarly, initial therapy with lipid formulations versus AmB-D did not affect prognosis (*p* > 0.05); this was attributed to the fact that almost all patients initially receiving AmB-D were subsequently switched to a lipid formulation. Combination therapy was administered to 25% of survivors compared to only 7.14% of non-survivors. Notably, the rate of combination therapy was significantly lower in the 12-week mortality group than in the survivor group (*p* = 0.025). However the choice of monotherapy versus combination therapy had no significant impact on overall mortality; combination therapy was used in 18.18% of the survivors and 15.09% of the non-survivors (*p* < 0.05). Regarding the rate of antifungal-related adverse events between survivors and non-survivors, adverse events were more frequent among the survivor group (12-week: 93.18% vs 59.54%, *p* < 0.001; overall: 93.94% vs 66.04%, *p* = 0.003).

No significant differences were found between patients receiving or not receiving antifungal prophylaxis, presence or absence of fungal co-infection, those with fungal co-infections treated with VRC or CAS in 12-week or overall mortality rates (*p* > 0.05).

There were no significant differences between surgery or non-surgery groups in 12-week mortality (43.75% vs 63.64%, *p* = 0.107). However overall mortality was 54.69% among surgically treated patients and 81.82% among those not operated on (p = 0.024).

Patients admitted to the ICU have a higher mortality rate compared to those who are not admitted (12-week: 77.78% vs 41.76%, *p* = 0.006; overall: 100% vs 51.47%, *p* < 0.001).

In univariable logistic regression analysis advanced age [12-week OR: 1.04 (95% CI: 1.01–1.07), *p* = 0.011; overall OR: 1.04 (95% CI: 1.01–1.07), *p* = 0.026 respectively], coexistence of both hematologic disease and DM [12-week OR: 5.73 (95% CI: 1.16–28.33), *p* = 0.032] associated with mortality. Surgical intervention was associated with a 73.2% reduction in the odds of overall mortality (OR: 0.268, 95% CI: 0.082–0.882, *p* = 0.030). Independent prognostic factors associated with mortality were analyzed using multivariable logistic regression analysis. Some variables (surgery, adverse effects, secondary infections) which occurred during treatment or hospitalization rather than at baseline were excluded from multivariable analysis to avoid bias. In the multivariable analysis, age was the only variable significantly associated with 12-week and overall mortality (respectively, *p* = 0.004 and *p* = 0.026). Each one-year increase in patient age was associated with a 1.05-fold increase (1.02–1.09) and 1.04-fold increase (1.01–1.07) in the odds of mortality, respectively.

## Discussion

This study, which includes 86 cases, represents the largest single-center series reported from Türkiye.

Mucormycosis can occur in all age groups but is rare in children [26, 27]. In studies of adult patients, the median age was reported as 51 years (IQR, 39–61) [14] and 48 years (min–max, 35–58.5) [15]. In our study, patients were between 20 and 86 years of age, with a median of 52 years (IQR, 42.5–62). Notably, the median age of deceased patients was significantly higher than that of survivors (56 vs. 48). Skiada et al., identified age as an independent risk factor for mortality [1]. Similarly, in a cross-sectional study of 550 patients in France reported that older age was significantly associated with poorer outcomes [7]. In our multivariable analysis, age was identified as an independent predictor of mortality. This increased odds in older individuals is likely due to a higher prevalence of comorbidities and reduced physiological resilience.

Mucormycosis is generally observed in tropical and subtropical climates [3, 13]. Reports from the Middle East have demonstrated that prevalence may vary according to season. Data on the seasonal distribution of *Mucorales* spores in the atmosphere are limited. Several case series from the Middle East have shown that the highest number of cases presented in autumn and summer [28, 29]. In a study from Spain, *Rhizopus* spp. were found to be more prevalent in the skin biota of dogs during summer, while *Mucor* spp. predominated in autumn [30]. In our study, mucormycosis cases most frequently presented in autumn (41.86%) and summer (36.05%). In a recent multicenter study from Türkiye, symptom onset was most prevalent in autumn (40.6%), while winter, spring, and summer showed comparable proportions (19.8% each) [31].

In the study of Skiada et al. [32] analyzing 382 cases, rino-orbito-cerebral (all sinus forms) was 36.6%, pulmonary 33.2%, and cutaneous 17.5%. In the study by Jeong et al. including 851 cases, sinus involvement was 34% (all sinus forms), pulmonary 20%, and cutaneous 22% [14]. In a Turkish series of 151 cases, 60% were rhinocerebral, 27.8% sinoorbital, and 4.6% pulmonary [33]. In our study, consistent with these reports, sinus involvement was the most frequent (77 cases, 89.53%), followed by pulmonary involvement (9 cases, 10.47%).

Studies reporting pulmonary mucormycosis as the predominant form generally included a higher proportion of hematologic malignancies [1, 2, 7, 20]. Conversely, in studies reporting rhinocerebral mucormycosis as the most common form, DM was the most frequent underlying factor (40%, 49%, 39%, respectively), followed by hematologic malignancy (33%, 39.7%, 31.4%, respectively) [14, 32, 33]. In our cohort, sinus involvement was 89.53%, despite hematologic disease being the predominant risk factor (59.3%), followed by DM (38.37%). In subgroup analysis, among patients with DM, sinus involvement was observed in 93.94% and pulmonary involvement in 6.06%. Among those with hematologic disease, sinus involvement was 90.2% and pulmonary involvement 9.8%. In patients with solid organ malignancies, sinus involvement was 57.14% and pulmonary involvement 42.86%. While pulmonary mucormycosis is typically more prevalent in cases of hematologic malignancy, our data showed a predominance of sinus involvement, consistent with another study conducted in Türkiye [34]. This discrepancy may stem from the late and nonspecific clinical presentation of pulmonary involvement, leading to delayed diagnosis or failure to detect the infection premortem [12, 34].

In our study the most frequent presenting symptoms were fever (70.93%), periorbital and facial swelling (51.16%), pain (47.70%), erythema (34.88%), nasal discharge (30.23%), and headache (25.58%). On physical examination, necrotic lesions of the oral cavity and sinuses (87.21%) and ocular findings (ophthalmoplegia, ptosis, vision loss, proptosis, anisocoria, chemosis) were the most common. No significant association was observed between mortality and any of the symptoms or physical examination findings. In a multicenter study from Türkiye, the most frequent symptoms were facial swelling, erythema, or pain (73.7%), followed by altered mental status (59.4%) and visual impairment (45.7%). The most common physical findings included erythema over the sinuses/orbit (73.7%), necrotic eschar in the nasal cavity (66.9%), and palatal eschar (59.4%). Altered mental status, visual impairment, lethargy, and proptosis were found to be associated with 90-day mortality [31].

Early diagnosis, initiation of treatment, and reduction of mortality are crucial, yet the diagnosis may not always be confirmed by microbiological and histopathological evidence [20, 35]. Roden et al. [12] reported culture positivity in 50% of biopsied patients, while Skiada et al. [32] found this rate to be 82%. A multicenter study in Türkiye reported a histopathological positivity rate of 84.2% and a culture positivity rate of 20.7% [31]. In our study, histopathological positivity was 79.73% and culture positivity was 62.83%. Differences may be related to sampling from areas with sparse hyphae, tissue crush artifact, or inadequate specimens (e.g., swabs, sputum), all of which can yield false negatives [8, 11, 35]. In addition, delayed sample transfer and technical issues can reduce diagnostic accuracy [42].

In cases where disseminated disease is suspected, a comprehensive diagnostic approach utilizing various clinical specimens is vital [6, 20]. Beyond tissue biopsy, the evaluation of respiratory secretions and BAL fluid through direct microscopy and culture remains essential, despite their limited sensitivity [1, 20]. To overcome these limitations, recent guidelines emphasize the role of *Mucorales*-specific real-time PCR in blood and respiratory samples for earlier detection. Furthermore, in patients with suspected CNS involvement, CSF sampling should be performed for culture and molecular analysis [6]. Finally, while galactomannan and beta-D-glucan are not produced by *Mucorales*, they play a critical role in the differential diagnosis by helping to rule out or identify co-infections with other invasive fungi [6, 20]. In our center, BAL sampling was performed in cases primarily to investigate suspected co-infections rather than dissemination, and no dissemination was detected in any of these patients. Additionally, CSF sampling was conducted in a limited number of cases, none of which showed findings suggestive of dissemination. Regarding fungal biomarkers, galactomannan was the only assay performed during the study period, and no other biomarkers were utilized.

Several studies have reported that the use of VRC increases the incidence of mucormycosis [36] and that the disease can develop in patients previously treated with CAS, VRC, FLC, ITC, or POS prophylaxis [1, 2]. Breakthrough infections have also been reported during ITC and POS prophylaxis [1, 14]. Skiada et al. also found that prior exposure to VRC and CAS was associated with a poor prognosis [1]. In our study, prior to the diagnosis of mucormycosis, 13.95% of patients had received FLC and 23.26% POS for prophylaxis; 4.65% VRC, 2.33% CAS, and 1.16% ITC for treatment. All five patients—all of whom had underlying hematologic malignancies—who received VRC, CAS, and/or ITC before the diagnosis of mucormycosis died. Furthermore, of the ten patients treated with VRC or CAS after the diagnosis of mucormycosis due to fungal co-infection, only three (30%) survived. Our observation of breakthrough mucormycosis following azole or echinocandin exposure aligns with existing literature, particularly regarding VRC. However, given the limited sample size of exposed patients in our cohort, these findings should be interpreted with caution and not be viewed as a definitive causal link. This observed high mortality rate in this subgroup is likely multifactorial. First, the majority of these cases were severely immunosuppressed patients with underlying hematologic malignancies, in whom the infection could not be fully controlled despite aggressive polyene therapy. Second, the virulence of the pathogen may have been enhanced under the selective pressure of VRC [37, 38]. Finally, the ‘antifungal pressure’ exerted by prior treatments likely led to significant delays in both diagnosis [20, 36] and the initiation of appropriate therapy [39], thereby further complicating the clinical outcome.

Lipid formulations of amphotericin B are recommended as initial therapy, followed by reassessment for step-down or maintenance based on clinical and radiological findings [6]. In a murine model comparing L-AmB with AmB-D, L-AmB was more effective and less toxic, while higher mortality was observed with AmB-D [40]. Skiada et al. reported mortality rates of 50% with AmB-D and ABLC compared with 32% with L-AmB [1]. In a Turkish study, clinical response rates were 56.8% for L-AmB and 21.4% for AmB-D [34].

In our study, the majority of patients received L-AmB at a standard daily dosage of 5 mg/kg, as recommended by international guidelines [6, 24]. However, clinical management frequently necessitated dose adjustments or temporary treatment interruptions due to significant adverse events, such as hypokalemia and renal impairment. Although we escalated the dosage to 7–10 mg/kg in only a limited number of cases, current global guidelines emphasize that higher dosages—specifically 10 mg/kg—should be considered, particularly for patients with CNS involvement, to achieve adequate drug penetration [6, 41]. Despite this recommendation, the risk of dose-dependent nephrotoxicity remains a significant challenge in real-world clinical practice [15, 41]. Our findings reflect that the management of treatment-related toxicities is a critical factor directly impacting therapeutic persistence. Ultimately, maintaining a delicate balance between aggressive dosing and cumulative toxicity management is vital to optimize outcomes in these high-mortality cases [1, 6, 41]. In the management of mucormycosis, POS and ISA play a critical role, particularly in patients with impaired renal function [6]. Marty et al. demonstrated in the VITAL study that ISA provides efficacy comparable to L-AmB and represents a safe alternative [42]. In addition, for refractory cases, recommended regimens include dose escalation of L-AmB, switching to ISA, switching to POS, or combination therapy with L-AmB and POS [6]. For maintenance therapy, ISA and POS are recommended due to their availability as oral formulations [6, 24]. In a European real-world study evaluating ISA for the treatment of mucormycosis, the drug was utilized as primary therapy in 5 cases and as salvage therapy in 12 cases out of 17 patients. The study reported a clinical response rate of 64.7% (n = 11) [43]. Since ISA is not available in our country, our center has no clinical experience with this agent. In our study, fourteen of our cases received L-AmB plus POS as salvage therapy, and eight of these patients died. The significantly lower prevalence of combination therapy among non-survivors at 12 weeks was linked to its role as a salvage intervention. Consequently, this variable was omitted from advanced statistical models to avoid potential immortal time bias.

In our study, adverse effects were observed in 76.7% (66/86) of patients. The most common were hypokalemia (72.1%, 62/86) and elevated urea–creatinine levels (28%, 24/86). In a Turkish study including 51 patients, hypokalemia (64.7%) and nephrotoxicity (45.1%) were the most frequent adverse effects [34]. In a prospective study on high-dose L-AmB, hypokalemia and a twofold increase in creatinine were the most frequent adverse events [41]. Our findings indicate that adverse effects were more frequent in survivors compared to the deceased patients. This observation highlights a paradoxical link between treatment complications and survival, which likely reflects treatment duration rather than a true protective effect. Specifically, patients with longer survived had extended exposure to antifungal therapy, thereby increasing their cumulative risk of developing side effects. Conversely, patients with early mortality may have died before these toxicities could manifest. Thus, the presence of adverse events in this cohort serves as a surrogate marker for longer treatment persistence.

Treatment is generally administered over several weeks or months; however, there is no standardized consensus. Duration should be individualized, continued until predisposing factors are corrected and clinical and radiological improvement is achieved [6, 8]. Oral posaconazole is recommended for maintenance therapy, with an evaluation of real-world data revealing a mean treatment duration of 6 months (range, 1 week–3 years) [6]. A multicenter study conducted in Türkiye reported a median total antifungal therapy duration of 28 days (IQR, 15.0–53.0) [31]. In our study, the median duration of amphotericin B therapy was 46.5 days (IQR, 14.7–84.5) and the median total duration of antifungal therapy was 46.5 days (IQR, 14.7–90.3). Among patients treated for ≤ 60 days, 81.13% died, 65.12% of whom had hematologic disease and 39.53% DM. Among patients who survived despite short treatment, early surgical intervention and localized sinus disease were common, suggesting that effective source control may allow for shorter antifungal courses in selected cases [6, 33].

Successful management of mucormycosis necessitates a multidisciplinary approach focused on early diagnosis, reversal of predisposing factors, prompt surgical debridement, and effective antifungal therapy [2, 6, 24]. In rhinocerebral mucormycosis, early surgical excision of infected sinuses and debridement increases survival rates and reduces the unnecessary loss of healthy tissues. In patients with pulmonary mucormycosis, the combination of surgical and antifungal therapy has been shown to improve survival compared with antifungal therapy alone [6]. In a recent study reported a 33.1% mortality rate with combined therapy versus 52.5% with antifungal agents alone [32]. The overall mortality rate was significantly lower in patients who underwent surgery (54.69%) compared to those managed with medical therapy alone (81.82%). In univariable logistic regression analysis surgical intervention was associated with a 73.2% reduction in the odds of overall mortality (OR: 0.268, 95% CI: 0.082–0.882, p = 0.030). However there were no significant differences between surgery or non-surgery groups in 12-week mortality (43.75% vs 63.64%). Consistent with our findings, data from a multicenter study in Türkiye indicated poorer survival among patients in the surgical group compared to the non-surgical cohort [31]. The lack of statistical significance in our model likely reflects the inherent selection bias typical of retrospective series. Patients with fulminant or rapidly progressive disease often reach a terminal state before appropriate surgical interventions can be performed. Consequently, this survival bias may confound the observed benefit in the surgical group, as those who underwent surgery might have had a more stable clinical course, allowing for operative intervention [15].

Mortality varies according to underlying disease, clinical form, and treatment, and has been reported as 40–80% [6]. A study in France reported that 90-day mortality rates were 55.8% in all patients, 64% in patients with hematological malignancies, 55.9% in SOT recipients, and 43.2% in those with DM [7]. Skiada et al. reported overall mortality of 47.8%, with 58.8% in hematologic malignancy/hematopoietic cell transplantation, 38.3% in DM, 39.3% with sinus involvement, and 54.6% with pulmonary involvement [32]. In our study, 12-week mortality was 48.84%, overall mortality was 61.63%. Also 12-week mortality rate was 54.9% in hematologic diseases (overall 62.75%), 54.55% in DM (overall 66.67%), and 42.86% in solid organ malignancy (overall 57.14%).

The observed association between ICU admission and mortality is interpreted as a reflection of disease progression. Since only the most critically ill patients are prioritized for ICU care, this variable functions as a marker of clinical severity. Therefore, it should be viewed as a proxy for multi-organ failure or severe inflammatory response rather than a discrete causative driver of poor outcomes. Furthermore, the poor prognosis associated with ICU admission may be compounded by the inherent risks of critical care, including secondary healthcare-associated infections and complications related to invasive mechanical ventilation or hemodynamic support.

Throughout the 21-year duration of our study, the diagnostic and therapeutic landscape for mucormycosis has evolved considerably [6, 7, 14, 20]. In recent years, the incidence of mucormycosis has increased due to the growing number of immunocompromised patients and advances in diagnostic methods [6, 14]. Despite this, improved diagnostic approaches, new antifungal agents, and combined medical-surgical treatments have increased survival [6, 7, 20]. Identification of risk groups and early suspicion remain critical [14, 15, 39]. The most common risk groups are patients with hematologic malignancy and uncontrolled DM [12, 14, 32]. Other risk factors include solid organ malignancy, BMT, SOT, corticosteroid therapy, deferoxamine, VRC use, intravenous drug use, trauma, burns [4, 12, 14, 44]. In some areas the epidemiology of mucormycosis has shifted significantly due to the emergence of CAM [44]. The meta-analysis by Özbek et al. demonstrated that the pandemic led to a global surge in rhino-orbito-cerebral cases [45]. Furthermore, Muthu et al. confirmed that uncontrolled diabetes and the inappropriate use of corticosteroids played a fundamental role in this outbreak [46]. Although CAM cases have been identified in our hospital, this study is limited to the pre-pandemic era.

Diagnosis has traditionally depended on conventional methods like direct microscopy and culture, which are unfortunately hindered by low sensitivity and often lead to critical delays in treatment [4, 20]. The introduction of advanced molecular systems, specifically *Mucorales*-specific real-time PCR in blood and respiratory specimens, represents a paradigm shift by facilitating earlier and more precise detection [6, 7, 35]. Similarly, while AmB-D has been abandoned and L-AmB remains the primary treatment [6, 41], the advent of next-generation azoles such as POS and ISA has broadened the options for both salvage and step-down therapy [6, 42]. Although our experience largely centers on conventional and liposomal formulations, incorporating these modern diagnostic and therapeutic innovations is essential to address the persistently high mortality rates of this disease [1, 6]. Novel agents are currently under development or clinical phase trial for cases resistant to existing therapies. In this context, fosmanogepix, which targets the fungal Gwt1 enzyme [40], and oteseconazole, a tetrazole antifungal, have demonstrated activity against certain *Rhizopus* species in both in vitro and in vivo studies [47].

Our study has several limitations. First, due to its retrospective design, the data are susceptible to selection bias, information bias, and missing clinical records. Second, the lack of PCR-based or other molecular genus- or species-level identification for *Mucorales* isolates limits the depth of our microbiological and epidemiological insights. Third, the study spans more than two decades, during which significant advancements in diagnostic techniques, antifungal therapeutics, and intensive care standards may have introduced temporal heterogeneity and affected data consistency. Finally, although surgical intervention appeared to be associated with lower mortality, this finding should be interpreted with caution, as it could not be fully adjusted for confounding by disease severity and may be subject to survivor bias.

To the best of our knowledge, this study represents the largest single-center cohort of mucormycosis reported from Türkiye to date. Beyond its scale, our study provides insight into the clinical management and outcomes observed over a 21-year study period. Notably, it introduces the observation that treatment-related adverse events may serve as a surrogate marker for therapeutic persistence—a finding that offers a unique clinical insight into the management of survivors requiring prolonged antifungal therapy. These findings contribute significant regional data to the global epidemiology of mucormycosis and underscore the persistent challenges in achieving early diagnosis and reducing high mortality rates.

In conclusion, our study demonstrates the high mortality associated with mucormycosis. In patients with DM, malignancy, or transplantation, advanced diagnostic work-up should be promptly undertaken once clinical suspicion arises, with the prompt initiation of antifungal treatment. Although mortality remains high, our results indicate that combining amphotericin B therapy with surgical intervention may reduce mortality.

## Data Availability

No datasets were generated or analysed during the current study.

## References

[1] Skiada A, Pagano L, Groll A, Zimmerli S, Dupont B, Lagrou K, et al. Zygomycosis in Europe: analysis of 230 cases accrued by the registry of the European Confederation of Medical Mycology (ECMM) Working Group on Zygomycosis between 2005 and 2007. Clin Microbiol Infect. 2011;17(12):1859–67. 10.1111/j.1469-0691.2010.03456.x. 21199154 10.1111/j.1469-0691.2010.03456.x

[2] Spellberg B, Edwards J Jr, Ibrahim A. Novel perspectives on mucormycosis: pathophysiology, presentation, and management. Clin Microbiol Rev. 2005;18(3):556–69. 10.1128/CMR.18.3.556-569.2005. 16020690 10.1128/CMR.18.3.556-569.2005 PMC1195964

[3] Prabhu RM, Patel R. Mucormycosis and entomophthoramycosis: a review of the clinical manifestations, diagnosis and treatment. Clin Microbiol Infect. 2004;10(Suppl 1):31–47. 10.1111/j.1470-9465.2004.00843.x. 14748801 10.1111/j.1470-9465.2004.00843.x

[4] Petrikkos G, Skiada A, Lortholary O, Roilides E, Walsh TJ, Kontoyiannis DP. Epidemiology and clinical manifestations of mucormycosis. Clin Infect Dis. 2012;54(Suppl 1):S23-34. 10.1093/cid/cir866. 22247442 10.1093/cid/cir866

[5] Bitar D, Van Cauteren D, Lanternier F, Dannaoui E, Che D, Dromer F, et al. Increasing incidence of zygomycosis (mucormycosis), France, 1997-2006. Emerg Infect Dis. 2009;15(9):1395–401. 10.3201/eid1509.090334. 19788806 10.3201/eid1509.090334 PMC2819884

[6] Cornely OA, Alastruey-Izquierdo A, Arenz D, Chen SCA, Dannaoui E, Hochhegger B, et al. Global guideline for the diagnosis and management of mucormycosis: an initiative of the European Confederation of Medical Mycology in cooperation with the Mycoses Study Group Education and Research Consortium. Lancet Infect Dis. 2019;19(12):e405–21. 10.1016/S1473-3099(19)30312-3. 31699664 10.1016/S1473-3099(19)30312-3 PMC8559573

[7] Gouzien L, Che D, Cassaing S, Lortholary O, Letscher-Bru V, Paccoud O, et al. Epidemiology and prognostic factors of mucormycosis in France (2012-2022): a cross-sectional study nested in a prospective surveillance programme. Lancet Reg Health Eur. 2024;45:101010. 10.1016/j.lanepe.2024.101010. 39220434 10.1016/j.lanepe.2024.101010 PMC11363841

[8] Riley TT, Muzny CA, Swiatlo E, Legendre DP. Breaking the mold: a review of mucormycosis and current pharmacological treatment options. Ann Pharmacother. 2016;50(9):747–57. 10.1177/1060028016655425. 27307416 10.1177/1060028016655425

[9] World Health Organization (WHO). Mucormycosis. https://www.who.int/india/home/emergencies/coronavirus-disease-(covid-19)/mucormycosis. Accessed 20 Mar 2026.

[10] Kontoyiannis DP, Lewis RE. Agents of Mucormycosis and Entomophthoramycosis. In: Blase MJ, Cohen JI, Holland SM, editors. Mandell, Douglas, and Bennett’s Principles and Practice of Infectious Diseases. Philadelphia: Elsevier; 2025. p. 3119–32.

[11] Ribes JA, Vanover-Sams CL, Baker DJ. Zygomycetes in human disease. Clin Microbiol Rev. 2000;13(2):236–301. 10.1128/CMR.13.2.236. 10756000 10.1128/cmr.13.2.236-301.2000 PMC100153

[12] Roden MM, Zaoutis TE, Buchanan WL, Knudsen TA, Sarkisova TA, Schaufele RL, et al. Epidemiology and outcome of zygomycosis: a review of 929 reported cases. Clin Infect Dis. 2005;41(5):634–53. 10.1086/432579. 16080086 10.1086/432579

[13] Chakrabarti A, Singh R. Mucormycosis in India: unique features. Mycoses. 2014;57(Suppl 3):85–90. 10.1111/myc.12243. 25187095 10.1111/myc.12243

[14] Jeong W, Keighley C, Wolfe R, Lee WL, Slavin MA, Kong DCM, et al. The epidemiology and clinical manifestations of mucormycosis: a systematic review and meta-analysis of case reports. Clin Microbiol Infect. 2019;25(1):26–34. 10.1016/j.cmi.2018.07.011. 30036666 10.1016/j.cmi.2018.07.011

[15] Patel A, Kaur H, Xess I, Michael JS, Savio J, Rudramurthy S, et al. A multicentre observational study on the epidemiology, risk factors, management and outcomes of mucormycosis in India. Clin Microbiol Infect. 2020;26(7):944.e9-944.e15. 10.1016/j.cmi.2019.11.021. 31811914 10.1016/j.cmi.2019.11.021

[16] Andresen D, Donaldson A, Choo L, Knox A, Klaassen M, Ursic C, et al. Multifocal cutaneous mucormycosis complicating polymicrobial wound infections in a tsunami survivor from Sri Lanka. Lancet. 2005;365(9462):876–8. 10.1016/S0140-6736(05)71046-1. 15752532 10.1016/S0140-6736(05)71046-1

[17] Neblett Fanfair R, Benedict K, Bos J, Bennett SD, Lo YC, Adebanjo T, et al. Necrotizing cutaneous mucormycosis after a tornado in Joplin, Missouri, in 2011. N Engl J Med. 2012;367(23):2214–25. 10.1056/NEJMoa1204781. 23215557 10.1056/NEJMoa1204781

[18] Warkentien T, Rodriguez C, Lloyd B, Wells J, Weintrob A, Dunne JR, et al. Invasive mold infections following combat-related injuries. Clin Infect Dis. 2012;55(11):1441–9. 10.1093/cid/cis749. 23042971 10.1093/cid/cis749 PMC3657499

[19] Rammaert B, Lanternier F, Zahar JR, Dannaoui E, Bougnoux ME, Lecuit M, et al. Healthcare-associated mucormycosis. Clin Infect Dis. 2012;54(Suppl 1):S44-54. 10.1093/cid/cir867. 22247444 10.1093/cid/cir867

[20] Skiada A, Lass-Floerl C, Klimko N, Ibrahim A, Roilides E, Petrikkos G. Challenges in the diagnosis and treatment of mucormycosis. Med Mycol. 2018;56(Suppl 1):S93-101. 10.1093/mmy/myx101. 10.1093/mmy/myx101 PMC6251532 29538730

[21] Ibrahim AS, Spellberg B, Avanessian V, Fu Y, Edwards JE Jr. *Rhizopus oryzae* adheres to, is phagocytosed by, and damages endothelial cells in vitro. Infect Immun. 2005;73(2):778–83. 10.1128/IAI.73.2.778-783.2005. 15664916 10.1128/IAI.73.2.778-783.2005 PMC547117

[22] Verweij PE, González GM, Wiederhold NP, Lass-Flörl C, Warn P, Heep M, et al. *In vitro* antifungal activity of isavuconazole against 345 Mucorales isolates collected at study centers in eight countries. J Chemother. 2009;21(3):272–81. 10.1179/joc.2009.21.3.272. 19567347 10.1179/joc.2009.21.3.272

[23] Vitale RG, de Hoog GS, Schwarz P, et al. Antifungal susceptibility and phylogeny of opportunistic members of the order *Mucorales*. J Clin Microbiol. 2012;50:66–75. 10.1128/jcm.06133-11. 22075600 10.1128/JCM.06133-11 PMC3256695

[24] Tissot F, Agrawal S, Pagano L, et al. ECIL-6 guidelines for the treatment of invasive candidiasis, aspergillosis and mucormycosis in leukemia and hematopoietic stem cell transplant patients. Haematologica. 2017;102(3):433–44. 28011902 10.3324/haematol.2016.152900 PMC5394968

[25] Donnelly JP, Chen SC, Kauffman CA, Steinbach WJ, Baddley JW, Verweij PE, et al. Revision and update of the consensus definitions of invasive fungal disease from the European Organization for Research and Treatment of Cancer and the Mycoses Study Group Education and Research Consortium. Clin Infect Dis. 2020;71(6):1367–76. 10.1093/cid/ciz1008. 31802125 10.1093/cid/ciz1008 PMC7486838

[26] Roilides E, Zaoutis TE, Walsh TJ. Invasive zygomycosis in neonates and children. Clin Microbiol Infect. 2009;15(Suppl 5):50–4. 10.1111/j.1469-0691.2009.02981.x. 19754758 10.1111/j.1469-0691.2009.02981.x

[27] Zaoutis TE, Roilides E, Chiou CC, Buchanan WL, Knudsen TA, Sarkisova TA, et al. Zygomycosis in children: a systematic review and analysis of reported cases. Pediatr Infect Dis J. 2007;26(8):723–7. 10.1097/INF.0b013e318062115c. 17848885 10.1097/INF.0b013e318062115c

[28] Al-Ajam MR, Bizri AR, Mokhbat J, Weedon J, Lutwick L. Mucormycosis in the Eastern Mediterranean: a seasonal disease. Epidemiol Infect. 2006;134(2):341–6. 10.1017/S0950268805004930. 16490139 10.1017/S0950268805004930 PMC2870385

[29] Talmi YP, Goldschmied-Reouven A, Bakon M, Barshack I, Wolf M, Horowitz Z, et al. Rhino-Orbital and Rhino-Orbito-Cerebral Mcormycosis. Otolaryngol Head Neck Surg. 2002;127(1):22–31. 10.1067/mhn.2002.126587. 12161726 10.1067/mhn.2002.126587

[30] Cabañes MFJ, Abarca ML, Bragulat MR, Castellà G. Seasonal study of the fungal biota of the fur of dogs. Mycopathologia. 1996;133(1):1–7. 10.1007/BF00437092. 8751821 10.1007/BF00437092

[31] Çiçek Y, Dumlu R, Koçak M, Pirdal BZ, Çelik M, Saltoğlu N, et al. Epidemiological profile and clinical outcomes of patients with mucormycosis: the multicenter retromucor study from Türkiye (2004–2024). Eur J Clin Microbiol Infect Dis. 2026;45:537–55. 10.1007/s10096-025-05397-x. 41575678 10.1007/s10096-025-05397-x PMC12987872

[32] Skiada A, Drogari-Apiranthitou M, Roilides E, Chander J, Khostelidi S, Klimko N, et al. A global analysis of cases of mucormycosis recorded in the European Confederation of Medical Mycology / International Society for Human and Animal Mycology (ECMM / ISHAM) Zygomyco.net Registry from 2009 to 2022. Mycopathologia. 2025;190(4):53. 10.1007/s11046-025-00954-6. 40493110 10.1007/s11046-025-00954-6

[33] Zeka AN, Taşbakan M, Pullukçu H, Sipahi OR, Yamazhan T, Arda B. Türkiye’den Bildirilen Zigomikoz Olgularının Havuz Analiz Yöntemi ile Değerlendirilmesi. Mikrobiyol Bul. 2013;47:708–16. 24237440 10.5578/mb.5836

[34] Kömür S, İnal AS, Kurtaran B, Ulu A, Uğuz A, Aksu HSZ, et al. Mucormycosis: a 10-year experience at a tertiary care center in Turkey. Turk J Med Sci. 2016;46(1):58–62. 10.3906/sag-1409-137. 27511334 10.3906/sag-1409-137

[35] Walsh TJ, Gamaletsou MN, McGinnis MR, Hayden RT, Kontoyiannis DP. Early clinical and laboratory diagnosis of invasive pulmonary, extrapulmonary, and disseminated mucormycosis (zygomycosis). Clin Infect Dis. 2012;54(Suppl 1):S55–60. 10.1093/cid/cir868. 22247446 10.1093/cid/cir868

[36] Marty FM, Cosimi LA, Baden LR. Breakthrough zygomycosis after voriconazole treatment in recipients of hematopoietic stem-cell transplants. N Engl J Med. 2004;350(9):950–2. 10.1056/NEJM200402263500923. 14985500 10.1056/NEJM200402263500923

[37] Lamaris GA, Ben-Ami R, Lewis RE, Chamilos G, Samonis G, Kontoyiannis DP. Increased virulence of Zygomycetes organisms following exposure to voriconazole: a study involving fly and murine models of zygomycosis. J Infect Dis. 2009;199(9):1399–406. 10.1086/597615. 19358672 10.1086/597615

[38] Lewis RE, Liao G, Wang W, Prince RA, Kontoyiannis DP. Voriconazole pre-exposure selects for breakthrough mucormycosis in a mixed model of *Aspergillus fumigatus-Rhizopus oryzae* pulmonary infection. Virulence. 2011;2(4):348–55. 10.4161/viru.2.4.17074. 21788730 10.4161/viru.2.4.17074

[39] Chamilos G, Lewis RE, Kontoyiannis DP. Delaying Amphotericin B-based frontline therapy significantly increases mortality among patients with hematologic malignancy who have zygomycosis. Clin Infect Dis. 2008;47(4):503–9. 10.1086/590004. 18611163 10.1086/590004

[40] Ibrahim AS, Avanessian V, Spellberg B, Edwards JE Jr. Liposomal Amphotericin B, and not Amphotericin B deoxycholate, improves survival of diabetic mice infected with *Rhizopus oryzae*. Antimicrob Agents Chemother. 2003;47(10):3343–4. 10.1128/aac.47.10.3343-3344.2003. 14506054 10.1128/AAC.47.10.3343-3344.2003 PMC201164

[41] Lanternier F, Poiree S, Elie C, Garcia-Hermoso D, Bakouboula P, Sitbon K, et al. Prospective pilot study of high-dose (10 mg/kg/day) liposomal Amphotericin B (L-AMB) for the initial treatment of mucormycosis. J Antimicrob Chemother. 2015;70(11):3116–23. 10.1093/jac/dkv236. 26316385 10.1093/jac/dkv236

[42] Marty FM, Ostrosky-Zeichner L, Cornely OA, Mullane KM, Perfect JR, Thompson GR 3rd, et al. Isavuconazole treatment for mucormycosis: a single-arm open-label trial and case-control analysis. Lancet Infect Dis. 2016;16(7):828–37. 10.1016/S1473-3099(16)00071-2. 26969258 10.1016/S1473-3099(16)00071-2

[43] Neofytos D, Pagliuca A, Houghton K, Broughton E, de Figueiredo Valente MLN, Jiang L, et al. Effectiveness, safety, and patterns of real-world isavuconazole use in Europe (2015-2019). Infect Dis Ther. 2024;13(12):2527–43. 10.1007/s40121-024-01064-4. 39443403 10.1007/s40121-024-01064-4 PMC11582280

[44] Muthu V, Rudramurty SM, Chakrabarti A, Agarwal R, et al. Epidemiology and pathophysiology of COVID-19-associated mucormycosis: India versus the rest of the world. Mycopathologia. 2021;186(6):739–54. 10.1007/s11046-021-00584-8. 34414555 10.1007/s11046-021-00584-8 PMC8375614

[45] Özbek L, Topçu U, Manay M, Esen BH, Bektas SN, Aydın S, et al. COVID-19–associated mucormycosis: a systematic review and meta-analysis of 958 cases. Clin Microbiol Infect. 2023;29(6):722–31. 10.1016/j.cmi.2023.03.008. 36921716 10.1016/j.cmi.2023.03.008 PMC10008766

[46] Muthu V, Agarwal R, Rudramurthy SM, Thangaraju D, Shevkani MR, Patel AK, et al. Multicenter case–control study of COVID-19–associated mucormycosis outbreak, India. Emerg Infect Dis. 2023;29(1):8–19. 10.3201/eid2901.220926. 36573628 10.3201/eid2901.220926 PMC9796192

[47] Hoenigl M, Sprute R, Egger M, Arestehfar A, Cornely OA, Krause R, et al. The antifungal pipeline: Fosmanogepix, Ibrexafungerp, Olorofim, Opelconazole, and Rezafungin. Drugs. 2021;81(15):1703–29. 10.1007/s40265-021-01611-0. 34626339 10.1007/s40265-021-01611-0 PMC8501344

